# Antibiotic Cement Utilization for the Prophylaxis and Treatment of Infections in Spine Surgery: Basic Science Principles and Rationale for Clinical Use

**DOI:** 10.3390/jcm11123481

**Published:** 2022-06-17

**Authors:** George M. Anderson, Camilo Osorio, Ellis M. Berns, Umar Masood, Daniel Alsoof, Christopher L. McDonald, Andrew S. Zhang, John Andrew Younghein, Eren O. Kuris, Albert Telfeian, Alan H. Daniels

**Affiliations:** 1 School of Medicine, Warren Alpert Medical School of Brown University, Providence, RI 02903, USA; george_anderson@brown.edu (G.M.A.); ellis_berns@brown.edu (E.M.B.); umar_masood@alumni.brown.edu (U.M.); 2 Department of Orthopaedic Surgery, Warren Alpert Medical School of Brown University, Providence, RI 02903, USA; camiloosorio608@gmail.com (C.O.); alsoofd@gmail.com (D.A.); christopher_mcdonald@brown.edu (C.L.M.); azhang@gwmail.gwu.edu (A.S.Z.); jandrew.younghein@gmail.com (J.A.Y.); eokuris@gmail.com (E.O.K.); 3 Department of Neurosurgery, Warren Alpert Medical School of Brown University, Providence, RI 02903, USA; atelfeian@lifespan.org

**Keywords:** antibiotic cement, surgical site infection, vertebral osteomyelitis

## Abstract

Antibiotic bone cement (ABC) is an effective tool for the prophylaxis and treatment of osteomyelitis due to the controlled, sustained release of local antibiotics. ABC has been proven to be effective in the orthopedic fields of arthroplasty and extremity trauma, but the adoption of ABC in spine surgery is limited. The characteristics of ABC make it an optimal solution for treating vertebral osteomyelitis (VO), a serious complication following spine surgery, typically caused by bacterial and sometimes fungal and parasitic pathogens. VO can be devastating, as infection can result in pathogenic biofilms on instrumentation that is dangerous to remove. New techniques, such as kyphoplasty and novel vertebroplasty methods, could amplify the potential of ABC in spine surgery. However, caution should be exercised when using ABC as there is some evidence of toxicity to patients and surgeons, antibiotic allergies, bone cement structural impairment, and possible development of antibiotic resistance. The purpose of this article is to describe the basic science of antibiotic cement utilization and review its usage in spine surgery.

## 1. Introduction

**Figure 1:**
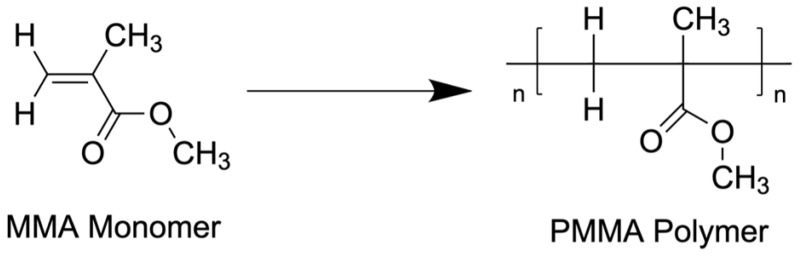
Molecular structure of monomethyl acrylate and polymethyl methacrylate polymer.

Antibiotic bone cement (ABC) has been widely used in orthopedic surgery for more than 150 years. ABC consists of polymethyl methacrylate acrylate (PMMA), an amorphous acrylate polymer, combined with one or multiple antibiotics, which can be introduced directly into a surgical wound (Figure 1). As such, ABC can deliver sustained high concentrations of antibiotics to a localized area. 

ABC has been shown to be efficacious for the prophylaxis and treatment of surgical site infections (SSI), particularly in the orthopedic subspecialities of arthroplasty and trauma [1,2,3,4]. In hip and knee arthroplasties, ABC prevents infection 90% more effectively than normal bone cement [5]. When compared to systemic antibiotic prophylaxis for preventing periprosthetic joint infection, utilizing ABC yielded lower infection rates and fewer systemic adverse effects. For instance, Josefsson et al. found that, two years after hip arthroplasty operations, patients who received systemic antibiotics had an infection rate of 1.6%, while those who received gentamicin-loaded bone cement had an infection rate of 0.4% [6]. Engesaeter et al. reported a 1.8-times higher rate of infection in primary hip replacement patients who received systemic antibiotics compared to those who received systemic antibiotics and gentamicin-loaded bone cement [7]. In the field of extremity trauma, Masquelet et al. described a surgical technique using ABC to treat large bone defects in open fractures. Utilizing this technique, an initial extensive debridement is performed, internal fixation achieved, and the residual void is filled with ABC. Six to eight weeks later, ABC is removed and replaced with a fresh autologous, cancellous bone graft [8]. A study of 27 open fractures treated by this technique resulted in zero residual infections [4]. 

Despite these established precedents, the field of spine surgery has been slow to adopt the use of ABC and there is a lack of literature on the use of ABC in spine surgery. This review seeks to explore common spinal infections and their pathogenesis, to describe the properties of ABC as it pertains to vertebral osteomyelitis (VO), and to summarize the current literature on ABC use in spine surgery.

## 2. Microbiology of Spinal Infections

VO refers to the infection and inflammation of vertebrae or intervertebral discs. VO accounts for 6% of all osseous infections [9]. It is more likely to develop in males (1.5 male to female ratio), is estimated to occur in 2.4/100,000 people in the general population, and incidence increases with age [10].

VO can cause a wide variety of symptoms, which may mimic degenerative spinal conditions, and time to diagnosis can vary from 11–59 days [11]. This delay allows pathogens to accumulate, spread, and compromise neural elements or render the spine structurally unstable. In such cases, eradication through medical treatment alone is less likely and surgical intervention is sometimes warranted. Despite surgical advances and new antimicrobial therapies, there may be substantial mortality and morbidity following VO [12,13].

**Figure 2:**
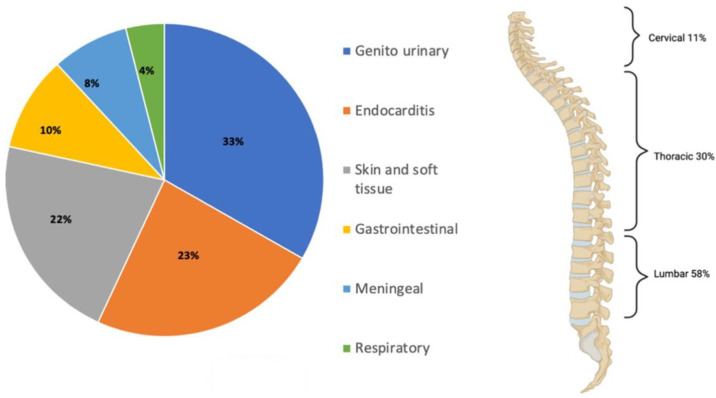
Sources of vertebral osteomyelitis and the most common spine segments affected.

The most common route of infection for VO is hematogenous spread, but VO may also originate from the genitourinary tract (17%), endocarditis (12%), skin and soft tissue infection (11%), gastrointestinal tract (5%), from the meninges (4%), and respiratory tract (2%). However, in up to 50% of all cases, the source cannot be identified [13]. VO preferentially affects the lumbar spine (58%), followed by the thoracic spine (30%) and then the cervical region (11%) [14] (Figure 2).

VO can also occur secondary to SSI [15]. Approximately 1–9% of all spine surgeries are complicated by SSI [16], and 0.7–11.9% of spine surgeries with instrumentation result in SSI [17]. SSI commonly occurs through direct contamination during the surgical procedure, typically from the patient’s endogenous skin flora, or sometimes from operating room personnel, hematogenous seeding, or early postoperative contamination [15,18].

### 2.1. Biofilms

**Figure 3:**
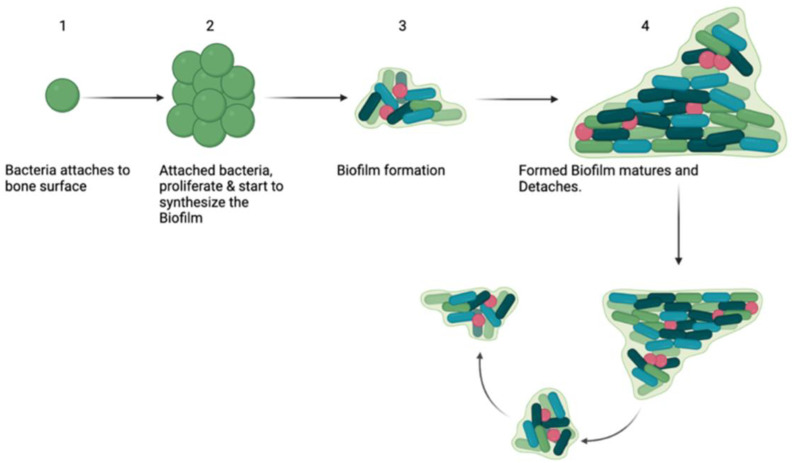
The process of biofilm formation.

Local bacteria can form a biofilm, a complex protective matrix of proteins, exopolysaccharides, and extracellular DNA. Bacteria in biofilms can be up to 10,000-times more resistant to antibiotics than free bacteria [19]. Biofilms contain pores that allow water and nutrient diffusion, as well horizontal gene transfer, conferring antibiotic resistance [20]. Within biofilms, bacteria communicate via quorum sensing to regulate matrix formation and show slowed metabolic growth and a diminished division rate due to limited oxygen and nutrition, making them insensitive to antibiotics that target rapidly dividing cells [20]. Biofilms form on the surfaces of instrumentation in five stages: attachment, preliminary matrix production, multiplication, maturity, and finally rupture and bacterial metastasis. [21,22] (Figure 3). Biofilm-compromised SSI can require hardware removal, resulting in a loss of correction of the spine or spinal instability [23]. In cases of spinal SSI, achieving high, sustained, local concentrations of antibiotics is paramount for the clearance and prevention of recurrent infection. 

### 2.2. Bacteria

**Figure 4:**
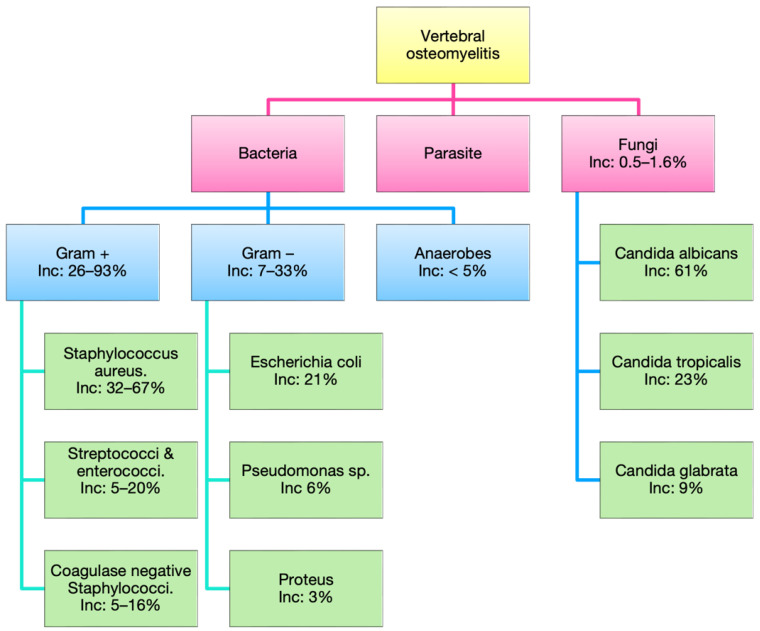
The different microbial causes of Vertebral Osteomyelitis (VO) and their rates.

VO is typically caused by bacterial pathogens, but, in rare cases, fungi and parasites are implicated (Figure 4). Infections are usually caused by a single microorganism (85%). Polymicrobial profiles have been reported in up to 9% of cases and are more common in patients with immunocompromise, diabetes, or intravenous drug abuse [13,14,22,23,24].

Gram-positive bacteria account for 26% to 93% of all bacterial cases, with *Staphylococcus aureus* (*S. aureus*) being the most frequently implicated, accounting for 32–67% of cases and 50% of cases of spinal SSI [13,14,15,24]. *S. aureus* is a Gram-positive cocci that asymptomatically colonizes approximately 20% of healthy individuals in the general population and up to 50–70% of healthcare workers [25]. The next most prevalent Gram-positive bacteria to cause VO are coagulase-negative staphylococci (CoNS), identified in up to 24% of cases [13,14].

Gram-negative bacteria are also implicated in cases of VO, albeit less frequently than Gram-positives. Enterobacteria spp. have been shown to be responsible for 7–33% of VO cases [14]. Of these, *Escherichia coli* (*E. coli*) is the most reported, at 21% of cases. Other less common Gram-negative bacteria that cause VO include *Proteus*, *Pseudomonas*, *Klebsiella*, and *Serratia* species, both of which are typical of urinary and gastrointestinal tract infections, especially in immunosuppressed and diabetic populations [26].

Mycobacterial infections can also cause atypical spine infections. In the past, *Mycobacterium tuberculosis* was implicated in up to 50% of cases, but today, this number is far lower, at least below 25% [14].

### 2.3. Fungi

Fungi are responsible for 0.5–1.6% of VO cases [27]. Risk factors for developing fungal VO are recent surgery, intravenous drug use, central venous catheters, and immunosuppression. The most common fungi implicated in VO are Candida species such as a *Candida albicans (C. albicans)*, which accounts for 61% of fungal VO [27]. *C. albicans* colonizes the skin and mucous membranes of healthy individuals [28]. Infections secondary to *C. albicans* are often associated with biofilm formation [29]. 

### 2.4. Parasites

Another possible cause of VO, though extremely rare in developed nations, is parasitic infection, due to ingestion of contaminated food or water. One such parasite is the protozoan *Balantidium coli*, which was reported in a recent, novel case of VO [30]. VO has also been observed due to spinal echinococcosis, or hydatid disease, from the *Echinococcus granulosus* parasite. Echinococcosis presents in bone in only 0.2–4% of cases, but of these, just over half are in the spine [31,32].

## 3. Fundamentals of Antibiotic Cement 

ABC is made up of one or multiple antibiotics combined with polymethyl methacrylate (PMMA) and a radiopacifier. PMMA is a hard, scratch- and shatter-resistant, amorphous acrylate polymer formed by mixing two components: a liquid monomethyl methacrylate (MMA) component and a powdered MMA component [33]. After these components are mixed, curing occurs. Curing time varies between different brands of ABC from five minutes to over 20 min, and it releases approximately 57 kJ per mol of energy, increasing the core temperature to approximately 77.3 degrees Celsius. Polymerization also increases viscosity and density. The viscosity of PMMA determines the working properties of the cement and increases from around 50 Pas to around 100 Pas. Theoretically, bone cement can shrink by approximately 6–7%; however, air inclusions in the cement dough limit shrinkage [34].

PMMA is available in several different formulations with their own unique qualities, many of which have commercially available versions containing antibiotics. These include Palacos R + G, Depuy CMW1, CMW2 and CMW3, Simplex P, Refobacin Bone Cement R, Cobalt HV, and Osteopal G. Osteopal G is geared specifically towards kyphoplasty and vertebroplasty, while the others are used mainly in arthroplasty (Table 1).

Antibiotics can also be added to PMMA by the surgeon. In this case, antibiotics are mixed into the MMA powder combination with the MMA liquid. This results in the incorporation of antibiotics between PMMA chains during the polymerization process [34]. After the antibiotics have been incorporated, they are released by reciprocal diffusion, which can be divided into two phases. The initial release is referred to as the “burst release” and occurs in minutes to hours. In this phase, high levels of antibiotic are released and diffuse into nearby tissue and fluids. The second phase, called “sustained release”, occurs after several days, resulting in a lower, but prolonged local antibiotic concentration [34].

The pharmacokinetic release profile of PMMA can be optimized. Each antibiotic has its own unique elution characteristics and combining multiple types of antibiotics can also increase elution. For example, Masri et al. showed that combining vancomycin and tobramycin in PMMA had a synergistic effect and caused vancomycin to release at higher concentrations for longer durations [35]. Adding polymeric fillers such as xylitol or glycine can also increase elution [34]. Furthermore, increasing the surface area increases elution as antibiotics release after contacting body fluids. Hand mixing PMMA, as opposed to vacuum mixing, results in a rougher and more porous surface, and therefore a higher surface area [34].

The intended use of ABC (for prophylaxis or treatment) and the susceptibility of the microorganisms identified or suspected determines the antibiotic choice. For prophylaxis, antibiotics should cover the most prevalent pathogens causing VO. Gentamicin, tobramycin, vancomycin, and clindamycin are the most widely used in ABC [36]. Aminoglycoside antibiotics such as gentamicin and tobramycin are effective against Gram-negative bacilli and tobramycin can also be used for some mycobacteria species. Many providers choose to use vancomycin, although there are some concerns with routine use for prophylaxis given the potential for antibiotic resistance [36,37]. Clindamycin is effective against anaerobic bacteria, Gram-positive cocci, and some atypical bacteria such as actinomyces [34]. 

Antibiotic combinations are also used in ABC, particularly when treating resistant infections. The efficacy of this strategy has been demonstrated both in vivo and in vitro. [38,39,40]. Combining tobramycin with vancomycin has been shown to be effective against a broad spectrum of bacteria, as well as against resistant species such as methicillin-resistant Staphylococcus aureus (MRSA). A combination of gentamycin and vancomycin has also been shown to be effective against MRSA. For cases of methicillin-resistant coagulase-negative Staphylococci, a combination of gentamycin, vancomycin, and clindamycin has been shown to be effective [41].

The ideal dose of antibiotic in ABC is a level high enough to inhibit bacterial growth for 3–4 weeks without inducing antibiotic resistance and a concentration low enough to avoid toxicity or structural compromise [42]. Depending on whether the goal is prophylaxis or treatment, different doses are required. For prophylaxis, ≤1 g antibiotic per 40 g of cement is recommended to avoid adverse structural effects, but this may be less important in spinal applications as compared to extremity joint applications [43]. For treatment of existing infections, higher doses are required for effective elution kinetics and for sustained therapeutic levels of local antibiotics [44]. In particular, 3.6 g of antibiotic per 40 g of cement has been suggested as an adequate dose for infection treatment as it is above the MIC for most microorganisms and limits structural compromise and potential toxicity [35].

## 4. Safety Concerns and Hazards

Concerns regarding ABC use include local toxicity, MMA vapor exposure, allergic response, structural compromise, inhibition of bone formation, damage to neurological structures, and the development of drug-resistant bacteria. There is limited evidence showing that ABC is toxic. However, an in vitro study by Edin et al. showed that vancomycin levels higher than >1000 ug/mL and tobramycin levels above >400 ug/mL decreased osteoblast replication, therefore interfering with bone homeostasis and possibly fusion. Ince et al. showed that osteoblasts exposed to high concentrations of gentamycin in vitro had reduced cellular viability and impaired bone production [45,46]. It is plausible that local antibiotics released in high concentrations from ABC could inhibit the functioning of osteoclasts and osteoblasts, and these results seem to support that vancomycin may be less locally toxic to osteoblasts than other antibiotics. Nonetheless, studies have demonstrated that the plasma concentrations of antibiotics eluted from bone cement are far below toxic thresholds. For example, Chohfi et al. demonstrated that for ABC used in hip arthroplasty, plasma levels were below 3 ug/mL, 30 times below the toxic threshold for vancomycin, and Kendoff et al. showed the mean postoperative maximum plasma concentration for gentamyciin ABC to be 2 ug/mL, six times below the toxic threshold [47,48].

MMA vapor exposure to members of the surgical team, particularly those who are pregnant, is also a concern. Exposure can cause respiratory, skin, and eye irritation, and can be toxic at levels higher than 1000 ppm [49]. In rodents, exposure to MMA vapor was shown to result in fetal and embryonic toxicity [50]. However, MMA vapor levels in the operating room are typically far below toxicity levels. A maximum exposure level of 100 ppm occurs during hip and knee arthroplasties [51]. 

Chronic exposure to MMA vapors is also implicated in cancer. A recent study reported that orthopedic surgeons exposed to MMA are more likely to die of cancer, particularly esophageal and myeloproliferative cancer, than general surgeons not exposed to MMA [52]. Using proper ventilation, vacuum mixing ABC instead of mixing by hand, and wearing protective headgear can limit potentially harmful MMA vapor exposure [53].

Cases of allergic reactions to ABC are uncommon and mostly related to the antibiotic being mixed, and so far, no special allergic precautions are deemed necessary. Evidence of ABC allergies includes a study by Thomas et al., which showed that 25 of 250 ABC arthroplasty patients with suspected allergies had reactions to gentamycin in ABC [54]. Further, a case report by Park et al. described a patient who developed drug fever after cement with piperacillin/tazobactam was loaded into her knee joint [55]. 

Some studies have shown that adding antibiotics to cement can reduce structural integrity. This detrimental effect increases with higher doses of antibiotics [56]. Lautenschlager et al. showed that adding more than 4.5 g per 40 g of ABC resulted in significantly decreased strength, and Lynch et al. showed an increased rate of mechanical failures when >3.6 g antibiotic per 40 g of cement was used compared to <1 g per 40 g of cement [57,58]. Furthermore, hand mixing was shown to decrease the mechanical strength of ABC compared to vacuum mixing, and liquid form antibiotics decreased mechanical strength more than powder form antibiotics [37,41,59]. Therefore, it is recommended to exercise caution and to plan for a possible decrease in mechanical strength when using antibiotic cement, to not exceed doses above 4 g of powdered antibiotic per 40 g cement, and to vacuum mix cement when possible.

There is also the potential that ABC itself could obstruct spinal fusion or damage the dura or other sensitive structures. Cement extravasation can occur following vertebroplasty and kyphoplasty, and in some cases, extravasation can lead to neurological damage [60,61]. Excluding cases of extravasation, no reports exist of stable ABC leading to neurovascular compromise. However, case reports exist of other implanted materials such as gel foam or cellulose leading to compression on nervous structures [62,63]. Surgeons must take care when applying ABC to encourage adequate fusion and to prevent friction on critical neurovascular structures.

ABC can provide a surface for bacterial colonization, and this, in addition to prolonged exposure to low doses of antibiotic, can cause antibiotic resistance. Kinnari et al. showed that, in general, the higher roughness of ABC leads to higher bacterial adhesion and a subsequent increase in antibiotic resistance [64]. Cement loaded with gentamycin has been shown to increase the resistance of coagulase-negative staphylococci (CoNS), *S. aureus*, *Staphylococcus multophilia*, and *Pseudomonas diminuta* in orthopedic revision surgery and of CoNS in a rat model [65,66,67].

## 5. Antibiotic Cement Use and Outcomes in Spine Surgery

Despite decades of proven use and study in orthopedic surgery, relatively little evidence exists regarding the efficacy of ABC in spine surgery. 

Two studies demonstrated the use of ABC for infection prophylaxis in spine surgery. Opalko et al. showed no cases of VO during one-year follow-up in 50 patients who underwent kyphoplasty supplemented with prophylactic ABC [68]. Kim et al. reported no SSI during 6-month follow-up in 10 cases where loose pedicle screws were revised and augmented with ABC [69].

Six studies demonstrated the use of ABC for the treatment of spinal SSI. Chen et al. described the successful eradication of a bacterial infection at T11 with the use of ABC vertebroplasty combined with an intravenous antibiotic regimen [70]. Masuda et al. successfully treated 11 patients with spinal SSI refractory to other treatments using ABC [71]. Ogihara et al. successfully treated three cases of deep SSI after cervical spine deformity surgery using ABC placed over and around the instrumentation [72]. Laratta et al. published a case series showing complete resolution of deep surgical site infections in ten spine surgery patients treated with permanent implantation of ABC over exposed instrumentation [73]. Lee et al. reported a case of a 63-year-old man with a staphylococcal spinal epidural abscess treated successfully with intravenous antibiotics and ABC beads introduced locally [74]. Slavnic et al. treated 62 patients with pyogenic spondylodiscitis of the thoracic spine with spinal reconstruction and fusion using antibiotic-impregnated PMMA. All patients achieved fusion and only one patient developed recurrent infection [75]. 

## 6. Conclusions

There is a significant potential benefit for more widespread usage of ABC in treating and preventing spinal VO. ABC can deliver high concentrations of antibiotics to a localized area, limiting the systemic effects of some antibiotics. If used in concert with systemic antibiotics, the rate of bacterial clearance could be optimized, recovery time shortened, and complications from chronic VO reduced. ABC also allows for sustained antibiotic elution, in some cases for up to 60 days, potentially reducing the percentage of VO relapse. ABC placed on or around instrumentation can also potentially decrease a wound’s dead space and enhance fixation strength, thereby reducing the risk of infection. 

New techniques could multiply the effectiveness of ABC in spine surgery. With the ongoing development and adoption of surgeries such as vertebroplasty and kyphoplasty, ABC can be delivered directly to the affected site, without the need for highly invasive procedures. This may decrease treatment duration and the amount of antibiotics used systemically.

However, despite these promising future directions, it remains vital to critically assess whether a patient requires ABC. It is important that ABC be used in accordance with its primary goal. When ABC is being used for prophylaxis, it must cover the most common causal pathogens and exceed the MIC for the suspected bacteria. When ABC is being used for treating an existing infection, the antibiotic must be specific for the causal pathogen, diminishing the possibility of bacterial resistance. Proper antibiotic-loading technique and dosage is essential to ensure mechanical strength and non-toxicity. Furthermore, removal of ABC in patients who fail initial treatment and need vertebral body resection may be challenging, and this risk should be weighed in each case. 

Even though ABC is not yet widely used in the field of spine surgery, it represents a valuable and theoretically effective alternative to current treatments of VO, which will become a more common pathology as the average age of the general population increases. Its use in spine surgery warrants further investigation.

## Figures and Tables

**Table 1:** Characteristics of widely used, commercially available brands of premixed antibiotic cement.

Brand	Radiopacifier	Color	Antibiotics Mixture	Setting Time and Temperature	Viscosity	Use
Palacos R + G bone cement	zirconium dioxide	green	0.5 g of gentamicin per 40.6 g	8 min, 45 s at 19 °C	high	arthroplasty
Depuy CMW1	barium sulfate	none	Optional: 1 g gentamicin per 40 g	12 min, 30 s at 19 °C	high	arthroplasty
Depuy CMW2	barium sulfate	none	Optional: 1 g gentamicin per 40 g	6 min, 30 s at 19 °C	high	arthroplasty
Depuy CMW3	barium sulfate	none	Optional: 1 g gentamicin per 40 g	12 min, 30 s at 19 °C	medium	arthroplasty
Simplex P	barium sulfate	none	Option 1 g tobramycin per 40 g	10 min at 19 °C	medium	arthroplasty
Refobacin Bone Cement R	zirconium dioxide	green	0.5 g gentamicin per 40 g	11 min at 19 °C	high	arthroplasty
Cobalt HV	zirconium dioxide	blue	Optional: 0.5 g gentamicin per 40 g	5 min at 23 °C	high	arthroplasty
Osteopal G	zirconium dioxide	green	0.325 g gentamicin per 26.53 g	23 min, 30 s at 20 °C	low	kyphoplasty and vertebroplasty

## Data Availability

Not applicable.
